# Supplementary material for: Striga hermonthica Suicidal Germination Activity of Potent Strigolactone Analogs: Evaluation from Laboratory Bioassays to Field Trials
Source: Plants (Basel). 2022 Apr 12;11(8):1045. doi: 10.3390/plants11081045 (PMC9025746; doi:10.3390/plants11081045)
Supplement: Supplementary file 1 [file plants-11-01045-s001.zip › plants-1654650-supplementary.pdf]

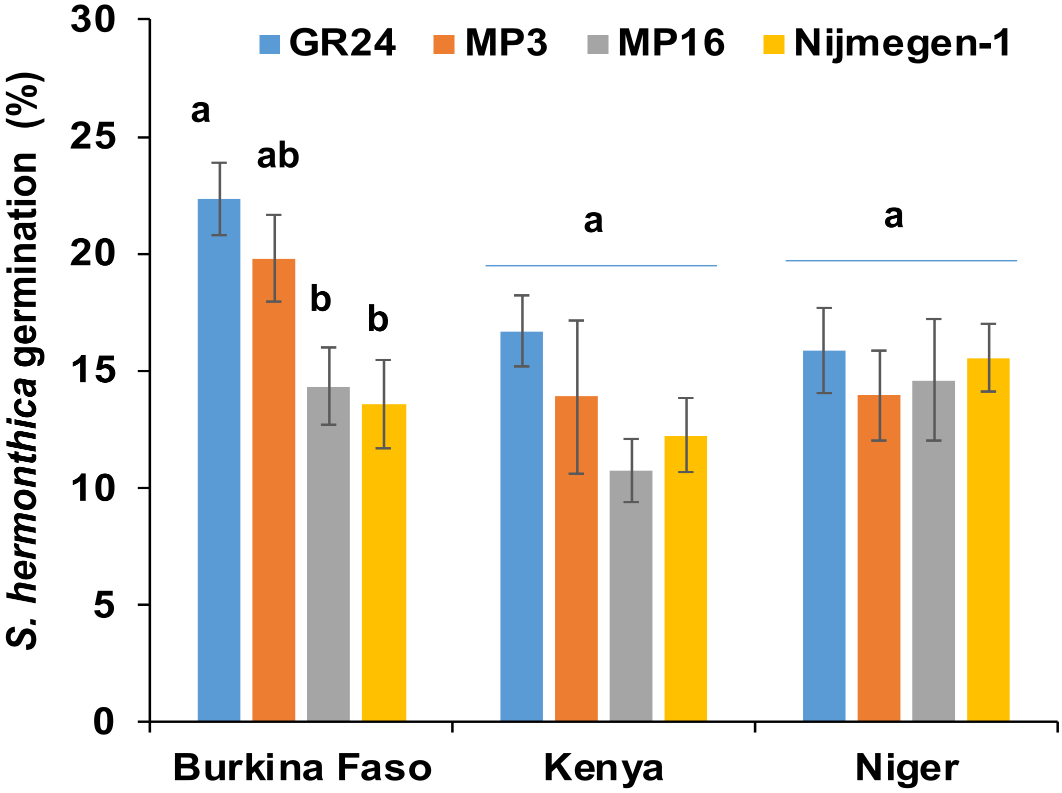

Figure S1. *Striga* seed germination in response to MP3, MP16, and Nijmegen-1 treatments. The selected SL analogs were applied at 1.0 µM concentration to the preconditioned *Striga* seeds collected from pearl millet fields in Burkina Faso and Niger, and a maize field in Kenya. GR24 was used as positive control. For each SL analog, treatments with various letters differ significantly according to one-way analysis of variance (ANOVA) and Tukey's post hoc test ($P < 0.05$). Error bars represent the standard error of the mean.
